# Comparison of the salivary and dentinal microbiome of children with severe-early childhood caries to the salivary microbiome of caries-free children

**DOI:** 10.1186/s12903-018-0693-1

**Published:** 2019-01-14

**Authors:** Eimear Hurley, Maurice P. J. Barrett, Martin Kinirons, Helen Whelton, C. Anthony Ryan, Catherine Stanton, Hugh M. B. Harris, Paul W. O’Toole

**Affiliations:** 1 School of Microbiology, University College Cork, Room 447 Food Science Building, Cork, Ireland; 2 APC Microbiome Ireland, University College Cork, Cork, Ireland; 3 Cork University Dental School & Hospital, Cork University Hospital, Wilton, Cork, Ireland; 4 Department of Neonatology, Cork University Maternity Hospital, Wilton, Cork, Ireland; 5 Teagasc Food Research Centre, Moorepark, Fermoy, Cork, Ireland; 6 College of Medicine & Health, University College Cork, Cork, Ireland

**Keywords:** Early childhood caries, Dentine, Saliva, Microbiota, Children

## Abstract

**Background:**

The main objectives of this study were to describe and compare the microbiota of 1) deep dentinal lesions of deciduous teeth of children affected with severe early childhood caries (S-ECC) and 2) the unstimulated saliva of these children and 3) the unstimulated saliva of caries-free children, and to compare microbiota compositional differences and diversity of taxa in these sampled sites.

**Methods:**

Children with S-ECC and without S-ECC were recruited. The saliva of all children with and without S-ECC was sampled along with the deep dentinal microbiota from children affected by S-ECC. The salivary microbiota of children affected by S-ECC (*n* = 68) was compared to that of caries-free children (*n* = 70), by Illumina MiSeq sequencing of 16S rRNA amplicons. Finally, the caries microbiota of deep dentinal lesions of those children with S-ECC was investigated.

**Results:**

Using two beta diversity metrics (Bray Curtis dissimilarity and UniFrac distance), the caries microbiota was found to be distinct from that of either of the saliva groups (caries-free & caries-active) when bacterial abundance was taken into account. However, when the comparison was made by measuring only presence and absence of bacterial taxa, all three microbiota types separated. While the alpha diversity of the caries microbiota was lowest, the diversity difference between the caries samples and saliva samples was statistically significant (*p* < 0.001). The major phyla of the caries active dentinal microbiota were Firmicutes (median abundance value 33.5%) and Bacteroidetes (23.2%), with *Neisseria* (10.3%) being the most abundant genus, followed by *Prevotella* (10%). The caries-active salivary microbiota was dominated by Proteobacteria (median abundance value 38.2%) and Bacteroidetes (27.8%) with the most abundant genus being *Neisseria* (16.3%), followed by *Porphyromonas* (9.5%). Caries microbiota samples were characterized by high relative abundance of *Streptococcus mutans, Prevotella* spp.*, Bifidobacterium* and *Scardovia* spp.

**Conclusions:**

Distinct differences between the caries microbiota and saliva microbiota were identified, with separation of both salivary groups (caries-active and caries-free) whereby rare taxa were highlighted. While the caries microbiota was less diverse than the salivary microbiota, the presence of these rare taxa could be the difference between health and disease in these children.

**Electronic supplementary material:**

The online version of this article (10.1186/s12903-018-0693-1) contains supplementary material, which is available to authorized users.

## Background

Dental caries is the “single most common chronic disease of childhood” [1] and affects 60–90% of all school children [2]. Severe early childhood caries (S-ECC) is an aggressive form of dental caries and is classified by the presence of a decayed, missing (due to caries), or filled tooth (dmft) index score of ≥4 (age 3), ≥5 (age 4), or ≥ 6 (age 5) [3]. S-ECC is destructive [4], and when it progresses it can cause acute pain and sepsis, and potential tooth loss [5]. Poor dental health in early childhood can interfere with the child’s quality of life, nutrition and school participation [4, 6]. Because of the young age of the children, S-ECC is difficult to treat successfully in the dental chair [7–9] and these children frequently require treatment under general anaesthesia [10] which increases treatment costs [11]. S-ECC is a risk factor for caries of permanent teeth [12–14] and affected children are at a higher risk of developing recurrent caries [8, 15, 16].

Dental caries has been investigated for many years using selective culture-based methods, and the role of *Streptococcus* and the presence of *Lactobacillus* have both long been recognized as playing substantial roles in dental caries [17–23]. Other non-microbiological risk factors that can play a significant role in S-ECC are host factors, environmental factors and diet [24, 25]. Dental caries is a product of loss of tooth structure and driven by acid produced by certain oral bacteria that ferment carbohydrate substrates, so diet plays a major role in the abundances of relevant microbes [26]. The impact of diet on the gut microbiome has been described [27, 28], but its impact on the oral cavity is not so well understood. Recent advances in molecular methods have allowed scientists to study the microbiology of oral disease with greater power, with technologies [29] such as 16S rRNA gene amplicon sequencing (including MiSeq and HiSeq platforms) showing that the microbiology of dental caries is much richer than previously believed.

The oral cavity has been shown to harbour more than 700 bacterial taxa, with one third of these described as non-culturable in vitro [30, 31], and two thirds belonging to cultivable species [32]. Although there is compositional variation between sample sites taken from the oral cavity, a ‘core’ microbiome in health has been identified [33]. Studies have also demonstrated that oral disease is not due to an isolated organism such as *Streptococcus mutans* causing caries, but is more polymicrobial in nature [23, 29–34]. Studies have identified *Bifidobacterium, Veillonella, Granulicatetta, Scardovia, Fusobacterium, Prevotella* and *Actinomyces* as potential contributors to ECC evidenced by their altered abundance in the caries microbiota [8, 12, 29, 30, 35–39].

To understand the microbiology of dental caries, it is helpful to use the combined findings from molecular and culture-based studies [37, 40–44] because molecular methods, when compared to culture-dependent methods have been shown to underestimate the proportions of certain phyla such as *Actinobacteria* [41, 45]. When examined in more detail, primer design was shown not to influence this bias [45]. 16S rRNA is highly conserved and the variable regions are high in G + C content. These high G + C regions in the DNA of *Actinobacteria* can result in an interruption of Taq polymerase processivity during the PCR process [41]. Primers used for this study targeted the V4 - V5 region, which has shown high classification accuracy when compared to other regional primer sites [46]. Studies have shown differences in the oral microbiota in children with caries and those that are caries free [21, 35, 37, 40]. *Streptococcus mutans* is found at higher levels at early stages of caries [47], while *Lactobacillus* sp. are associated with disease progression of caries and *Scardovia* sp. have been isolated from dentinal caries and have previously been associated with having a role in the advancement of deep caries in S-ECC [40, 41, 48, 49].

Limited data are available by molecular methods on the microbiota of advanced deep dentinal caries and pulpal infections of deciduous teeth, knowledge of which could aid in the development of anti-bacterial medicaments in pulp therapy of these affected teeth. Gram-negative species have been identified in deep dentinal caries of ECC affected teeth [43, 44], and are present in deep pulpal infections of primary teeth [50–55]. The microbiota of exposed vital pulp chambers of carious deciduous teeth was found to be dominated by phyla Firmicutes and Actinobacteria using anaerobic culture and by comparison with taxa in the Human Oral Microbiome Database [56]. Rôças et al. [57] using Illumina Miseq sequencing identified these same phyla to dominate the microbiota of deep dentinal caries of permanent teeth with irreversible pulpitis.

In the present study, Illumina Miseq sequencing was employed to compare the microbiota of the deep dentinal lesions of S-ECC affected deciduous teeth, and saliva of these caries-active children, with the saliva of caries-free children. S-ECC is an aggressive form of caries, and we analysed the microbiota of the deep dentinal caries of deciduous teeth to determine if the salivary microbiota was a reservoir or source of taxa linked with this form of caries. The main objectives were to describe and compare the microbiota of 1) deep dentinal lesions of deciduous teeth of children affected with S-ECC and 2) the unstimulated saliva of these children and finally, 3) the unstimulated saliva of caries-free children and compared compositional differences and diversity of taxa in these sampled sites.

## Materials and methods

### Study design, ethics and recruitment

The study design was to recruit two cohorts of children under the age of 60 months. These two groups were categorized into those with S-ECC (caries-active), and a caries-free cohort, all medically healthy. Sample size calculations were estimated based on previous similar studies [36, 47, 58, 59]. In total, we recruited 68 caries-active and 70 caries-free children. The deep dentinal lesion microbiota were labelled as Caries-active cavity (CAC) and the salivary microbiota of these caries-active children labelled as Caries-active saliva (CAS), while the saliva of the caries-free children were labelled as Caries-free saliva (CFS). Of the caries-active, all were S-ECC affected deciduous teeth, and the CAC and CAS are paired samples, each from the same subject. Ethical approval was obtained from the Teaching Hospitals Clinical Research Ethics Committee (Cork, Ireland) for the recruitment and sampling of these Cohorts of children.

Recruiting of the children affected by S-ECC was performed at a Hospital Dental Treatment Centre. All children were referred to the Hospital Dental Treatment Centre (Cork, Ireland) where clinical examinations were performed and they were then scheduled for extraction of their carious teeth, under general anaesthetic. All these children referred to the Hospital Dental Treatment Centre for general anaesthetic had radiographs taken as part of the examination prior to referral. The caries-free cohort was recruited from various crèches, where a paediatric dentist travelled to each of the crèches and examined the children’s teeth.

Inclusion criteria applied to both caries-free and caries-affected groups were that they were medically healthy, had no antibiotic intake in the 3 months prior to sampling, and were under the age of 60 months.

### Diet and lifestyle data collection

Before undergoing the dental examination, informed consent was obtained from the parent/guardians of the children. Habitual dietary data was also collected using a validated Food Frequency Questionnaire (FFQ) [27] which was provided to each parent/guardian, to record food intake (Additional file 1). A detailed questionnaire was given to each parent/guardian. Data collected included antibiotic treatment history, general medical history and dental history, fluoridation status of home (well/public water), feeding practices in infancy, birth mode, and oral health related quality of life.

### Oral examination

Oral examinations for both caries-free and caries-active groups were performed by a trained Paediatric dentist after a full dental examination was completed by a dental surgeon in the clinic. Children in crèches and in the Hospital Dental Treatment Centre were examined in a quiet area with their parents present. Children’s teeth were wiped with a cotton wool roll and sterile gauze square to remove plaque and debris prior to examination which was carried out under natural light, using a standard size 4 mirror and ball ended CPI ‘C’ probe [60]. The mouth was illuminated with a Promed Penlight, which consists of bright concentrated halogen light when natural light was insufficient. For the caries-active group, caries was recorded at the level of cavitation into dentine (cavitation level), using the WHO criteria [60, 61], with the addition of visible non cavitated dentine caries as referenced by Whelton et al. [62]. The International Caries and Detection Assessment System (ICDAS) code for the caries affected teeth were within codes 5 and 6 [63]. The dmft score was recorded along with the dmfs score, and sample collection performed. For the caries-free group, caries was recorded at the level of cavitation into dentine (cavitation level), using the WHO criteria [61]. The dmft/dmfs was measured, and sample collection was performed. Caries-free children did not show clinical evidence of early pre-cavitation of caries or white spot lesions and had no history of treatment on any tooth surfaces, as defined [64].

### Sampling

The same-trained paediatric dentist took all samples after the teeth were examined. All children were instructed not to brush their teeth the evening and the morning before sampling. A CatchAll™ collection swab, with hard pack for storage after collection was used (Cambio UK) [65] (See image: Additional file 2). For the caries-active S-ECC group, both a carious lesion sample and a saliva sample were taken. After a full dental examination, and pre-general anaesthetic, the CatchAll™ collection swab was used to collect pooled unstimulated saliva in the floor of the mouth for 1–2 min. This unstimulated saliva sample is recognized as a representation of the whole oral ecosystem [33, 66, 67]. The swab was placed back in the collection tube, and stored at − 80 °C. To sample the carious lesions, while the child was under general anaesthetic, the carious deciduous tooth was extracted and under isolation, the tooth was irrigated with saline. Under care, by a paediatric dentist, the superficial carious dentin was excavated with a sterile spoon excavator and the next layer of deep dentinal caries was excavated using a new separate sterile spoon excavator and the sample was pooled in a sterile 1.5-ml micro-centrifuge tube with 1 ml of TE buffer (50 Mm Tris-HCL, 1 Mm EDTA). The samples were placed in a sterile 1.5-ml micro-centrifuge tube and transported to the laboratory, where they were frozen until further analysis and stored at − 80 °C. For the caries-free group, after a full dental examination, the CatchAll™ collection swab was used to collect pooled unstimulated saliva in the floor of the mouth for 1–2 min. The swab was placed back in the collection tube, and stored at − 80 °C.

#### DNA extraction

Extraction of DNA from all samples was carried out with the MO BIO PowerLyzer® 24 homogenizer following some initial optimisation for extraction from an oral catch-all swab rather than a soil sample as previously described [68]. The saliva sample was contained in a catch-all swab in the end of a collection tube. The tube was cut 1 cm above this swab, and this was inserted into the PowerBead tubes, to which 60 μl of solution C1 had been added. Tubes were incubated at 65 °C for 10 min and then shaken horizontally at maximum speed for 2 min, using the MO BIO vortex adapter. The remainder of the protocol was followed as per manufacturer’s instructions. For the caries sample, the tubes were incubated at 65 °C for 10 min and then shaken horizontally at maximum speed for 4 min, using the MO BIO vortex adapter. The remainder of the protocol was followed as per manufacturer’s instructions. DNA was visualised on a 0.8% agarose gel and quantified using the Nanodrop 1000 (Thermo Scientific, Ireland). DNA was then stored at − 80 °C.

#### 16 s rRNA gene amplification primers

Primers used for PCR amplification were the V4 - V5 region primers 520F (AYTGGGYDTAAAGNG) and 926R (CCGTCAATTYYTTTRAGTTT)**.** Initial primers for Illumina sequencing contain the sequencing primer binding sites, forward or reverse 16S rRNA gene specific primer, and a 10 nt in-line multiplexing identifier (MID). Dual separate MIDs were attached to both ends of the PCR product.

The V4 - V5 amplicons for Illumina sequencing were generated using a two-step amplification procedure. The first step reaction mix contained 50 μl BIO-X-ACT™ Short Mix (BIOLINE), 10 μl of 2 nM forward and reverse primers, 50 ng genomic DNA, and ddH_2_0 to give a final volume of 100 μl. Cycling conditions were: an initial 95 °C, 5-min denaturation step; 30 cycles of 95 °C for 15 s, 42 °C for 15 s, and 72 °C for 30s; and a final 10-min extension at 72 °C. The products were purified using SPRIselect beads (Beckman Coulter, Indianapolis IN) as per manufacturer’s instructions, using a 0.9:1 volume ratio of beads to product. The purified PCR products were eluted in 40 μl of ddH_2_0. DNA quantity was assessed via Quant-iT™ PicoGreen® dsDNA Assay Kit (Invitrogen™). The samples were pooled in equimolar amounts and then sequenced by Eurofins Genomics (Eurofins Genetic Services Ltd., I54 Business Park, Valiant way Wolverhampton WV9 5GB, UK) using Illumina MiSeq 2 × 300 bp paired end technology. Nextflex Rapid library preparation was carried out by the company to attach bridge adaptors necessary for clustering. Sequencing of 16S DNA was carried out on the V4/V5 region using a Miseq (301 bp paired-end reads). Sequence data were stored on a Linux server and backed up on external hard-drives.

### Bioinformatic analysis

#### Sequence processing, OTU clustering and taxonomy assignment

The software, flash (v1.2.8), was used to join paired-end reads. Paired-end reads with more than 25% incorrect bases in their region of overlap were excluded from subsequent steps. Qiime (v1.9.1) was used to extract barcodes (extract_barcodes.py) and for demultiplexing (split_libraries_fastq.py).

The USEARCH (v8.0.1623) pipeline was used for the following steps: de-replication of reads (identical reads are represented by a single sequence), exclusion of reads shorter than 350 bp and longer than 370 bp, exclusion of unique reads, chimera filtering, OTU clustering at 97% identity and calculation of representative OTU sequences. Using USEARCH, all reads (including unique reads) were then mapped back to the representative OTU sequences to give the final OTU read count for each sample. The software fastQC (v0.11.3) was used after each filtering step to assess read quality. The median read count for the samples after sequence processing was 44,400. The sample number after sequence processing was 206.

Part of the mothur (v1.36.1) [69] pipeline was used to run the RDP classifier using a filtered version of the RDP database in order to assign taxonomy down to genus level. The software SPINGO (v1.3) [70], was used to assign taxonomy at species level. For both mothur/RDP and SPINGO, confidence cut-offs of 80% were used.

#### Alpha and beta diversity analysis

Alpha and beta diversity metrics were calculated in Qiime (v1.9.1) [71]. To calculate diversity metrics, several additional steps were carried out (also in Qiime). The OTU table was rarefied (single_rarefaction.py) at 10,540 reads (the lowest read count in the dataset). Representative OTU sequences were aligned using pyNAST (align_seqs.py) and filtered to remove columns that do not contribute to phylogenetic signal (filter_alignment.py). A phylogenetic tree was generated using FastTree (make_phylogeny.py). This tree is necessary for phylogenetic alpha and beta diversity metrics. The rarefied OTU table was used in the calculation of all diversity metrics.

The following alpha diversity metrics were calculated: chao1, Shannon (Shannon’s index), Simpson (Simpson’s index), Observed species (OTU count) and Phylogenetic (PD whole tree). The following beta diversity metrics were calculated: weighted and un-weighted unifrac distances, and Bray-Curtis dissimilarity.

#### Statistics and data visualisation

All statistics and data visualisation were carried out in R (v3.2.3) [72]. Paired Mann-Whitney tests were used to compare microbiota of saliva samples (CAS) (*n* = 68) with that of caries samples (CAC) (*n* = 68) taken from the caries group. Both CAC and CAS are paired since both samples from the same subject. Un-paired Mann-Whitney tests were used to compare the saliva and caries samples from the caries group with a control group of caries-free saliva (CFS) (*n* = 70) of caries-free individuals. Benjamini and Hochberg correction [73] was used to adjust *p*-values for multiple testing. Level of significance was set at *p* < 0.05.

## Results

### A distinct microbiota in caries lesions but not saliva in children with S-ECC

**Fig. 1:**
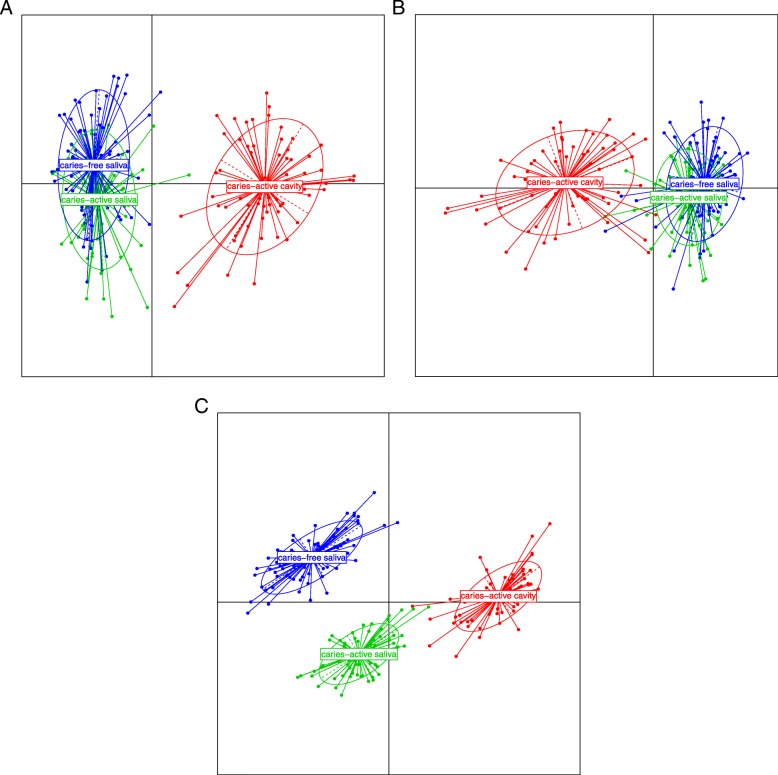
PCoA (principle co-ordinates) plots showing relatedness by two established metrics, Bray Curtis dissimilarity, and UniFrac distances, while unweighted UniFrac illustrates separation between the three groups (CAC, CAS & CFS). **a**. Plot of principle co-ordinates using Bray-Curtis dissimilarity. Points are coloured according to group and ellipses describe the distribution of points for each group. Percentage variation explained: PCA 1 (22.3%) and PCA 2 (7.7%). **b**. Plot of principle co-ordinates using weighted unifrac distance. Points are coloured according to group and ellipses describe the distribution of points for each group. Percentage variation explained: PCA 1 (45%) and PCA 2 (11.7%). **c**. Plot of principle co-ordinates using un-weighted unifrac distance. Points are coloured according to group and ellipses describe the distribution of points for each group. Percentage variation explained: PCA 1 (16.6%) and PCA 2 (4.9%)

Given that S-ECC is such an acute disease, it seemed possible that it resulted from a global microbiota change in the oral cavity. To investigate the relatedness of microbiome composition in CAC (caries-active caries), CAS (caries-active saliva) and CFS (caries-free saliva), we generated PCoA (principle co-ordinates) plots showing relatedness by two established metrics, Bray Curtis dissimilarity, and UniFrac distances. The Bray Curtis plot (Fig. 1a) shows separation of the caries lesion samples (CAC) and the two saliva sample types (CFS & CAS) based on PCoA axes 1 and 2. The microbiota of the two saliva groups (CAS & CFS) considerably overlap, indicating a similarity in the general composition of microbial taxa. For weighted UniFrac, (Fig. 1b) the caries microbiota group is again separated from the two saliva microbiota groups, with only minimal overlap of some samples. Combined with the Bray Curtis analysis, this shows convincingly that there is no major separation between the microbiota of CAS and CFS groups, even though this metric is very sensitive to the differences in the presence/absence and abundance of OTUs/samples.

Plotting the second UniFrac metric, unweighted UniFrac distances (Fig. 1c), illustrates separation between the three groups (CAC, CAS & CFS). This index measures the presence and absence of taxa only and does not adjust the distance metric according to taxon abundance, so unlike weighted analysis, it reflects the contribution of rare taxa (that get overwhelmed in a weighted analysis). When presence/absence of taxa is the dominant parameter used to calculate distance, separation between all three groups occurs. The CAS microbiota was closer to the CAC microbiota than the CFS microbiota, suggesting rare taxa are shared between the former two samples.

### Microbiota diversity in caries lesions is lower than that of saliva from children with or without S-ECC

**Fig. 2:**
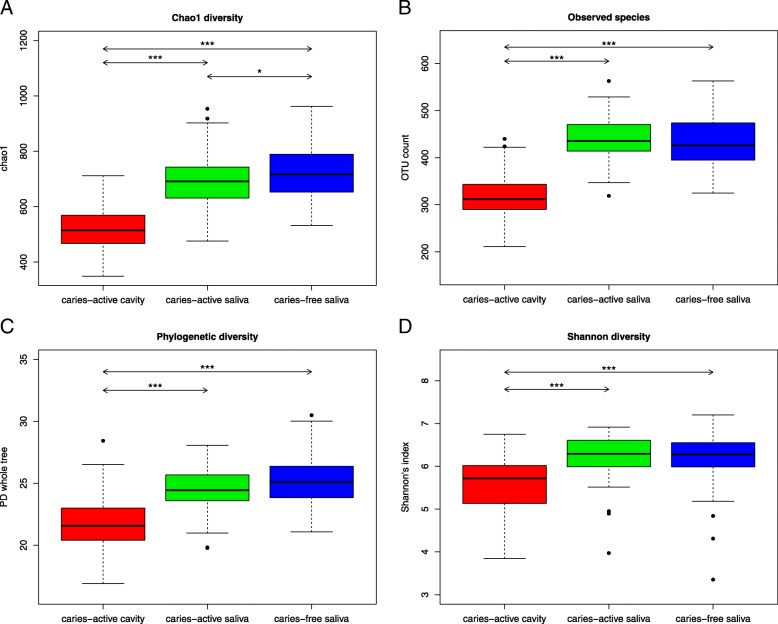
Pairwise alpha diversity comparisons of saliva and caries microbiota. **a**. Boxplot of chao1 diversity in the three groups. Outliers are represented by black points. Significant differences between groups are shown by arrows and the following notation: *p* < 0.05 (*), *p* < 0.01 (**) and *p* < 0.001 (***). **b**. Boxplot of observed species in the three groups. Outliers are represented by black points. Significant differences between groups are shown by arrows and the following notation: *p* < 0.05 (*), *p* < 0.01 (**) and *p* < 0.001 (***). **c**. Boxplot of Phylogenetic diversity in the three groups. Outliers are represented by black points. Significant differences between groups are shown by arrows and the following notation: *p* < 0.05 (*), *p* < 0.01 (**) and *p* < 0.001 (***). **d**. Boxplot of Shannon diversity in the three groups. Outliers are represented by black points. Significant differences between groups are shown by arrows and the following notation: *p* < 0.05 (*), *p* < 0.01 (**) and *p* < 0.001 (***)

To study the diversity of the microbiota from the caries lesions (CAC) and saliva samples of both caries-free children (CFS) and caries-active children (CAS), a series of alpha diversity metrics was determined: the Chao index, phylogenetic diversity (PD whole tree), observed species (OTU count), the Simpson index and the Shannon index (Fig. 2). All metrics illustrate that the diversity of caries microbiota was the lowest of these sample types. The difference in the diversity values of the caries samples and saliva samples was significant (*p* < 0.001). The Chao diversity measurement, as illustrated in Fig. 2a, estimates the number of species from observed data, and the diversity of the low abundance taxa, and according to this metric, the CFS versus CAS comparison was significantly different with a *p*-value of < 0.05. All other alpha diversity comparisons (Fig. 2b, c, d) indicated that the diversity difference between the caries samples and saliva samples was significant (*p* < 0.001).

### Habitual diet is not significantly different in children with or without S-ECC

Diet can have a profound impact on oral health and caries risk. We found minor technical inconsistencies within the FFQ recording of data between patients and this could have a negative impact on the significance of diet and its frequency on the oral microbiota of these children. With the data that was recorded (Additional file 3), no significant differences were found when the habitual intake of each food group was compared from the FFQ data derived from the caries-active and caries-free subjects. No food group was consumed at significantly different frequency when we tested either for unequal presence/absence of food groups in the diet using the Fisher’s test, or different frequencies of food groups in the diet using the Mann-Whitney test. Any trends were not supported by significant *p*-values (< 0.05).

### Differentially abundant taxa in CAC compared to saliva microbiota of both CAS & CFS

**Fig. 3:**
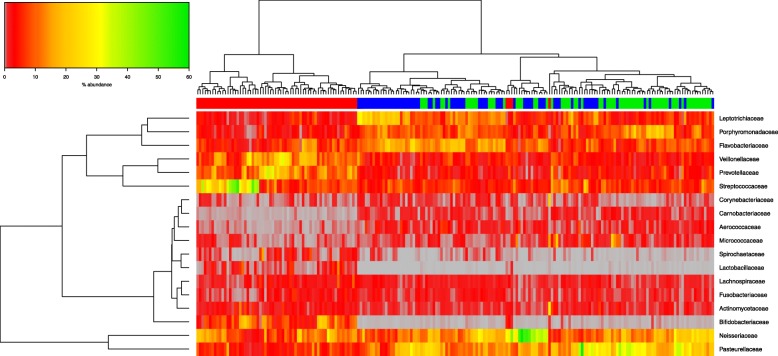
Hierarchical clustering of microbiota data at bacterial family level. Abundances are colour-coded according to the colour key on the top left with grey representing a value of zero. Euclidean distance and complete linkage were used to cluster the rows and columns of the heatmap. The colour bar on top of the heatmap corresponds to sample type: CAC red, CAS green and CFS blue. All taxa present at less than 1% in all three groups are excluded from the heatmap

Differential bacterial taxon abundance in compared microbiota datasets may be graphically demonstrated by hierarchical clustering, whereby samples are grouped based on similarity of the taxa in their microbiota. These relatedness levels between samples, and their constituent microbial taxa, are represented by vertical and horizontal dendrograms incorporating a “heatmap” colour scale to convey abundance levels. Figure 3 illustrates the abundance of bacterial taxa at family level. The three groups are split into two main branches visible in the horizontal dendrogram above the colour bar in Fig. 3. CAC clusters on one branch (red bar) and the two saliva groups (CAS in green & CFS in blue) cluster on the other. There is clustering of the CAS and the CFS within this branch, showing considerable similarity between the two groups at family level. When compared against the patient metadata, there was no obvious variable that convincingly separates CAS from CFS (data not shown).

The microbiota of the CAC samples was characterized by high relative abundance of *Prevotellaceae, Veillonellaceae, Bifidobacteriacae and Streptococcaceae*, and by low relative abundance of *Corynebacteriaceae, Carnobacteriaceae, Aerococcaceae,* and *Micrococcaceae.* Both saliva sample types (CAS & CFS) showed higher abundances than caries samples of *Leptotrichiaceae, Porphyromonadaceae and Flavobacteriaceae* and of *Neisseriaceae* and *Pasteurellaceae*, while illustrating a very low abundance compared to CAC of *Spirochaetaceae, Bifidobacteriaceae and Lactobacillaceae.*

**Fig. 4:**
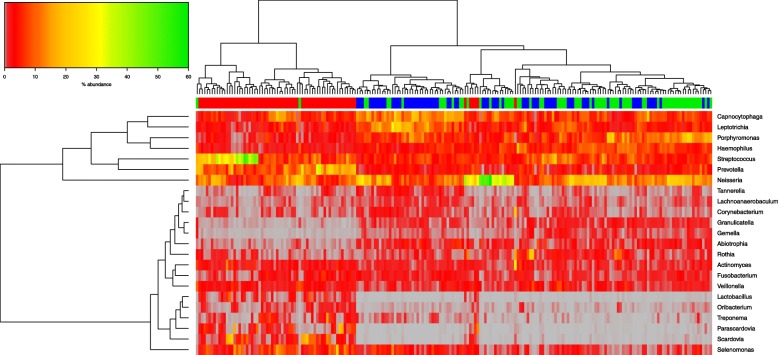
Hierarchical clustering of microbiota data at bacterial genus level. Abundances are colour-coded according to the colour key on the top left with grey representing a value of zero. Euclidean distance and complete linkage were used to cluster the rows and columns of the heatmap. The colour bar on top of the heatmap is coloured according to sample type: CAC red, CAS green and CFS blue. All taxa present at less than 1% in all three groups are excluded from the heatmap

A more nuanced picture emerges when differentially abundant taxa were analysed at genus level (Fig. 4). The samples again separate laterally into caries versus saliva (with both caries-active and caries-free clustering together). The microbiota content appears to be split vertically in two groups of differentially abundant genera as revealed by the dendogram on the Y-axis based on bacterial abundance. The top branch is divided, with CAS and CAC showing higher abundance of *Streptococcus* and *Prevotella*, and with *Neisseria* at a higher abundance in both. In the saliva samples (CAS & CFS), *Leptotrichia, Porphyromonas and Haemophilus* are in higher abundance, with *Leptotrichia* at higher abundance in CFS than CAS.

The lower branch shows clear low-abundance of the genera *Lactobacillus, Treponema, Scardovia,* and *Parascardovia* in the CFS & CAS*.* (Fig. 4), while low abundance taxa in CAC are *Gemella* and *Granulicatella*.

When samples in the analysis with at least one species with a median value of ≥0.5% were included, fewer outliers were identified, with only one CAS sample within the CAC branch, and two CAC within the second branch of the saliva samples. There was clearer separation of the samples, with CAC on the first branch, with high abundance of *Streptococcus mutans*, compared to the saliva samples. The two saliva samples split into two branch points, with CFS illustrating clustering within the first branch, with higher abundance of *Tannerella forsythia, Capnocytophaga gingivalis*, and *Leptotrichia buccalis.* Species with a clear low abundance in the majority of saliva samples included *Scardovia wiggsiae, Parascardovia denticolens, Prevotella denticola* and *Prevotella oris*, where these were present at higher abundance in CAC.

### Broad and fine detail compositional differences distinguish caries microbiota from paired and healthy control saliva samples

**Fig. 5:**
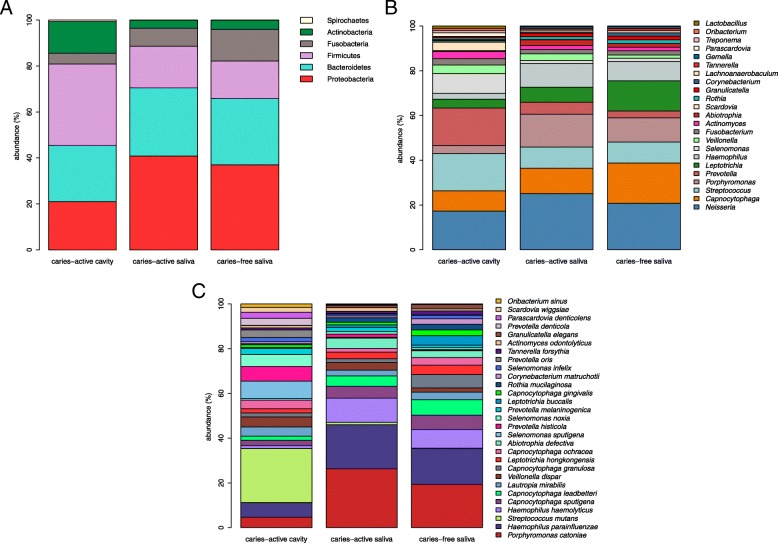
Broad and fine detail compositional differences at Genus, phylum and species level. **a**. Microbiota composition at phylum level. Percentages for each taxon represent the median abundance values for the sample types. **b**. Barplot of percentage abundance at genus level. Percentages for each taxon represent the median values for the groups. **c**. Barplot of percentage abundance at species level. Percentages for each taxon represent the median values for the groups

At phylum level (Fig. 5a), the CAC microbiota was dominated by Firmicutes (median abundance value 33.45%), while the CAS microbiota was dominated by Proteobacteria (median abundance value 38.18%; *p* < 0.0001). At phylum level, both the CAS and CFS microbiota composition is quite similar (Fig. 5a), with CAS and CFS microbiota dominated by Proteobacteria at 38.2 and 36.1% respectively. The main phylum difference between saliva of CAS versus CFS children was Fusobacteria. Its abundance has a median value of 13.4% in the saliva microbiota of CFS children, but 7.37% in CAS (*p* < 0.0001).

**Fig. 6:**
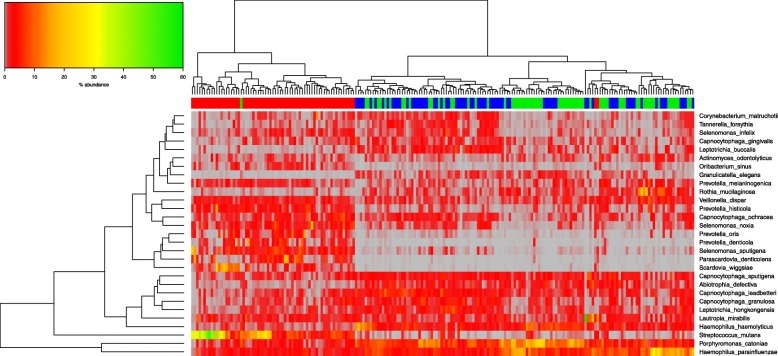
Hierarchical clustering of microbiota data at bacterial species level. Abundances are colour-coded according to the colour key on the top left with grey representing a value of zero. Euclidean distance and complete linkage were used to cluster the rows and columns of the heatmap. The colour bar on top of the heatmap is coloured according to sample type: CAC red, CAS green and CFS blue. All taxa present with at least one species with a median value ≥0.5% in all three groups are included

Excluding the “other” and “unassigned” categories, the three microbiota types (CAC, CFS & CAS) are dominated by taxa *Neisseria, Capnocytophaga, Porphyromonas, Streptococcus, Prevotella, Leptotrichia,* and *Haemophilus. Streptococcus, Neisseria, Prevotella, Capnocytophaga,* dominate the CAC sample microbiota and at lower levels, taxa *Scardovia, Parascardovia, Selenomonas* and *Lactobacillus*. CAC sample microbiota include numerous species of higher relative abundance: *Streptococcus mutans, Alloprevotella denticola, Prevotella histicola, Scardovia wiggsiae, Parascardovia denticolens, Prevotella tannerae* and *Bifidobacterium dentium.*

The CFS microbiota composition differs significantly from the saliva of CAS group by the presence of the following microbial genera: *Leptotrichia, Bifidobacterium, Corynebacterium, Alloprevotella, Cardiobacterium* and *Veillonella* (*p* < 0.0001). The abundance of all six genera was significantly higher in CFS (*p* < 0.0001).

The CAS microbiota was dominated by *Neisseria, Porphyromonas, Streptococcus* and *Haemophilus,* and species included *Streptococcus mutans, Prevotella histicola, Prevotella melaninogenica, Porphyromonas catoniae* and *Prevotella salivae*. CFS samples were dominated by *Leptotrichia, Capnocytophaga, Neisseria, Haemophilus, Streptococcus* and *Porphyromonas* at genus level*,* while at species level, the CFS group include species *Haemophilus haemolyticus, Haemophilus parainfluenzae, Rothia mucilaginosa, Porphyromonas catoniae* and *Streptococcus sanguinis.*

Microbial taxa showing statistically significant differential abundance between CFS and CAS children included *Streptococcus mutans, Haemophilus parainfluenzae, Prevotella histicola, Leptotrichia buccalis, Veillonella dispar, Alloprevotella tannerae* and *Prevotella salivae.* Interestingly, *Streptococcus mutans, Prevotella histicola* and *Veillonella dispar* were present at higher abundance in CAS than the saliva of CFS.

## Discussion

In this study, next generation sequencing of 16S amplicons was used to explore the microbiota of deep dentinal carious lesions and saliva of Irish children affected with S-ECC and the salivary microbiota of those that are caries-free.

Using two well established metrics (Bray Curtis dissimilarity and UniFrac distance) the caries dentine microbiota was found to be distinct from that of either CFS or CAS, illustrating, when abundance is taken into account, that CAC has considerably different proportions of certain high-abundance taxa. However, when measured using unweighted UniFrac, which measures presence and absence of taxa only, all three groups were found to be distinct, showing that each group is different in terms of rare or low-abundance taxa. Of interest was the closer microbiota relatedness of CAS to CAC, with the presence of certain CAS outliers overlapping with CAC. This suggests that some samples share similar low-abundance taxa between CAC and CAS or that CAS harbours some taxa which may have stimulated the increased caries rate compared to CFS, or there is some shedding of taxa from CAC into the CAS. When a leave-one-out strategy was implemented with dominant taxa such as *Neisseria* and *Streptococcus*, these outliers changed position, sometimes clustering within their own group and a few samples that clustered within their group in the full dataset became outliers with the reduced dataset. When these outliers were studied in detail, these samples were found not to be influenced by any metadata, suggesting that factors not accounted for in this study are responsible for unusual taxon composition in a subset of samples.

Focusing on the dominant taxa and their abundance illustrated graphically by hierarchical clustering on the heatmap, samples were identified and grouped based on microbiota similarity, but also of interest was the partial agreement between the outliers identifiable in the PCoA plots and in the heatmaps. The identity of these outliers could not be readily correlated with patient metadata and when the taxon abundance level was investigated in detail, ar genus and family levels ther is splitting of CAC with both the saliva groups (CFS & CAS), with the CFS samples clustering mostly together, with some intermixing of both saliva microbiota types. There is clear clustering of CAC in branch one to left, with CFS at next branch in blue (Figs. 3, 4), with CAS lastly split on a third branch. This is more apparent at species level by hierarchical clustering, with less intermixing of both salivary microbiota types (Fig. 6).

This incomplete separation of samples between saliva groups suggests that the salivary microbiota is not specific enough to be used as an identifier for caries risk in children. The oral cavity is an entry point for colonisation of microbial species and saliva is a reservoir for a multitude of bacteria, with its microbial and nutritional composition being shaped by food intake, reflux, environment and other influences [38, 67, 74]. In this study, no association was found between factors like habitual diet, brushing habits or fluid intake with microbiota composition, although it is possible these metadata are not sufficiently granular in the FFQ data and patient questionnaire. Furthermore, previous studies have shown variability between sites in the oral cavity itself, with niches among the tongue, soft and hard palates, supra- and sub-gingival surfaces of teeth and saliva each demonstrating microbiota variability [32, 66]. The flow rate, buffering capacity, and molecules within saliva which can aid attachment of bacterial cells, all play a role in both the compositional balance of the oral microbiome [75]. While some studies have also found an association between the microbiota and disease in plaque samples, but not within the saliva samples [37, 76] our findings support these findings, with saliva and caries representing two distinct habitats.

As caries lesions progress and become more severe, the diversity of caries microbiota decreases [34, 35, 47]. In this study, alpha diversity of caries microbiota was lowest, and differences in the diversity of the caries samples and saliva samples were significant (*p* < 0.001). However the Chao diversity index for CFS was significantly higher than CAS with a *p*-value of < 0.05, again supporting the previous data, that when low-abundance or rare taxa are given an equal weighting to higher-abundance taxa, differences in diversity between the two saliva groups become apparent. This suggests that even at low abundance, certain taxa such as the acid-producing lactobacilli can play a strong role in caries progression. This highlights an important possibility, that taxon abundance of cavity-causing microbes may not be strongly correlated with progression of caries; low-abundance taxa at abundance levels that typically do not feature in microbiome summary data might be the main indicator of future tooth decay because, for instance, a small number of acid-producing or biofilm-producing species may have a disproportionate impact on oral health. In addition, the potential affect that the removal or exclusion of these rare taxa may have on caries prevention and general oral health makes a solid case for their identification.

As caries progresses to a more advanced state, the bacteria that dominate this cavity are less diverse, because aciduric organisms have been selected and enriched, and we found that the main genera that dominated the CAC lesion were *Neisseria, Streptococcus* and *Prevotella*, while the species that dominate the caries lesion (CAC) include *Streptococcus mutans, Prevotella sp, Scardovia sp.* and *Bifidobacterium dentium*. *Neisseria, Streptococcus, Prevotella* and *Porphyromonas* have all been strongly associated with caries in past studies [29, 35, 38, 40, 41, 44, 58]. *Neisseria* and *Streptococcus* produce acid which lowers the pH of the mouth and leads to increased demineralisation of enamel [47] while *Prevotella* has a known role in caries progression and endodontic infections [7, 50]. The high level of *Streptococcus mutans* in the carious lesion is consistent with previous studies, and its presence is a strong indicator for caries [58]. *Streptococcus mutans* aids in caries initiation by adhering to the enamel, forming a cariogenic biofilm via glucan synthesis on the surface, aiding the binding of other species. While being aciduric and acidogenic, it can often be part of a more complex community of microorganisms working together [40, 44, 47, 77], and is a risk factor for caries progression [34].

*Scardovia* is documented as having a role as a cariogenic bacterium involved in the later stages of S-ECC [40]. *Scardovia wiggsiae* is significantly associated with S-ECC, based on a culture study of plaque from children [36], and in adults with caries [78]. *Prevotella* species have been shown to play an important role in endodontic infections [50], and *Prevotella tannerae*, *Prevotella histicola* (isolated from human oral mucosa [37]) and *Alloprevotella denticola* [41, 74] have all been shown to be associated with dental caries. *Lactobacillus*, which is notably associated with caries progression [22, 39, 44, 48, 79] was found at very low levels compared to other genera (0.675% (CAC) and 0.031% (CAS)). These low levels were also reported in previous studies [34, 38, 47] and this interesting finding supports the idea that when certain acid producers are at low levels, their acidogenic properties may nevertheless be strong enough to allow other acid producers to take their place, such as *Neisseria*, *Selenomonas* and *Streptococcus mitis* [47]. *Neisseria* spp. have the ability to metabolize glucose to produce lactic acid and this genus was found at high levels in CAC (10.29%) and in CAS (16.28%) and may have an active cariogenic role. *Lactobacillus* is found at low levels in endodontic infections with deep caries [80, 81]. It has also been suggested by Rôças et al. that altered *Lactobacillus* abundance may be due to the change from cariogenic microbiota to a microbiota that stimulates progression into pulpal tissue causing infection. Shifts in the microbiota composition at the outermost pulpal layer can be affected by saliva and diet to the outermost pulpal layer, while the inner deeper layer has a different environment in comparison [57]. The replacement of *Lactobacillus* with other taxa could be linked to the degree of pain, duration of pain, length of caries destruction, connection with pulp, diet and environmental factors, and this fine detail could reveal reasons for the low levels of *Lactobacillus* in these teeth.

Within CFS there were higher levels of *Capnocytophaga* (10.9%) and *Leptotrichia* (8.1% CFS), and lower levels of *Porphyromonas* and *Neisseria*. There is evidence to suggest that *Capnocytophaga* and *Leptotrichia* are health-associated species [36, 44, 58, 77] and *Capnocytophaga* has been found at higher levels in caries-free subjects [37, 47, 58]. Lower levels of *Porphyromonas* were found (9.5% CAS & 6.6% CFS) and *Neisseria* (16.3% CAS & 12.6% CFS) in CFS compared to CAS. Some *Neisseria* sp. have been shown to play a role in acid production (e.g. *N. gonorrhoeae* and *N. meningitidis*) [47], while other *Neisseria* species such as *Neisseria flavescens* have been shown to elicit higher signal of probes when targeted in caries-free children [67] together with the *Porphyromonas* gram-negative species, *Porphyromonas catoniae.* The association of these species with a caries-free oral status has been further supported by Nyvad et al. [29]. A higher abundance of *Porphyromonas catoniae* was found within the CAS (9.5%)*.* Studies have reported children with high levels of caries harbouring higher levels of *Porphyromonas* in their saliva [78], by culture study [40], and it has been detected in root canals of necrotic deciduous teeth [79] and our findings demonstrate a positive association of *Porphyromonas* with S-ECC.

## Conclusion

In conclusion, we identified distinct differences between the caries microbiota and saliva microbiota, with separation of both salivary groups (caries-active and caries-free), showing a clear separation when low abundance taxa were considered. While the microbiota diversity in the caries dentinal microbiota was lower than both salivary microbiota types, there were clear compositional differences between all groups from phylum to species. Firmicutes dominated the CAC, while Proteobacteria dominated the CAS and CFS salivary microbiota, and genera that dominated the CAC lesion were *Neisseria, Streptococcus* and *Prevotella*. The taxa present in the dentinal lesions could be potential instigators that drive migration of infection to the pulp, while the saliva microbiota in health and disease may be associated with caries-active or caries-free status in these children.

## Additional files

Additional file 1: Food Frequency Questionnaire (FFQ) template used. (XLS 48 kb)

Additional file 2: Image of Catch-all™ swab used for the collection of oral saliva samples. (DOCX 18 kb)

Additional file 3: Data collected from FFQ. (XLSX 775 kb)

## Abbreviations

BLAST: Basic local alignment search tool
CAC: Caries-active caries
CAS: Caries-active saliva
CFS: Caries-free saliva
dmfs: Decayed, missing (due to caries), or filled tooth surfaces in primary teeth
dmft: Decayed, missing (due to caries), or filled tooth in primary teeth
ECC: Early childhood caries
FFQ: Food frequency questionnaire
FLASH: Fast Length Adjustment of SHort reads to improve genome assemblies
MID: Multiplexing identifier
OTU: Operational taxonomic unit
PCoA plot: Principle co-ordinates plot
QIIME: Quantitative Insights Into Microbial Ecology
rRNA: ribosomal Ribonucleic acid
S- ECC: Severe Early childhood caries
