# Supplementary material for: Comparison of the salivary and dentinal microbiome of children with severe-early childhood caries to the salivary microbiome of caries-free children
Source: BMC Oral Health. 2019 Jan 14;19:13. doi: 10.1186/s12903-018-0693-1 (PMC6332856; doi:10.1186/s12903-018-0693-1)
Supplement: Supplementary file 2 — Image of Catch-all™ swab used for the collection of oral saliva samples. (DOCX 18 kb) [file 12903_2018_693_MOESM2_ESM.docx]

[
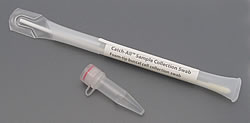
](https://www.google.ie/url?sa=i&rct=j&q=&esrc=s&source=images&cd=&ved=2ahUKEwi30rvK5a3eAhXEBsAKHSBpAgYQjRx6BAgBEAU&url=http://wyszukiwarka.biocena.pl/zestawy-do-izolowania-dna/603-epicentre-buccalamp-dna-extraction-kit-with-catch-all-soft-pack-buccal-swab-bq0916ssc-1.html&psig=AOvVaw2QQA3w4KHZ3wBffLZpuK7u&ust=1540976132556993)

Additional file 2: CatchAll^TM^ Collection swab Image. (Cambio UK)
